# Outcomes Following Antifungal Treatment for *Candida* Growth in Bile Cultures Collected During Endoscopic Retrograde Cholangiopancreatography

**DOI:** 10.3390/jof12030208

**Published:** 2026-03-14

**Authors:** Grace Charpentier, Kevin Andrew Smith, James E. Slaven, Theresa O. Emeli, Rachel G. Susler, Hamed Chehab, Mark A. Gromski, Haseeba Khan, Samir K. Gupta, Nicolas Barros

**Affiliations:** 1 Indiana University School of Medicine, Indianapolis, IN 46202, USA; 2 Division of Infectious Diseases, Department of Medicine, Indiana University School of Medicine, Indianapolis, IN 46202, USAsgupta1@iu.edu (S.K.G.); 3 Department of Biostatistics and Health Data Science, Indiana University School of Medicine, Indianapolis, IN 46202, USA; 4 Department of Medicine, Indiana University School of Medicine, Indianapolis, IN 46202, USA; temeli@iu.edu (T.O.E.);; 5 Division of Gastroenterology and Hepatology, Department of Medicine, Indiana University School of Medicine, Indianapolis, IN 46202, USA; 6 Department of Pathology and Laboratory Medicine, Indiana University School of Medicine, Indianapolis, IN 46202, USA; khanha@iu.edu

**Keywords:** bile cultures, invasive candidiasis, biliary candidiasis, endoscopic retrograde cholangiopancreatography

## Abstract

*Candida* species are frequently detected in bile cultures during endoscopic retrograde cholangiopancreatography (ERCP), but their clinical significance and the value of antifungal treatment remain unclear. We performed a retrospective single-center cohort study of adults with growth of *Candida* species from bile cultures collected by ERCP performed between 2010 and 2023. We compared inpatients who received vs. those who did not receive antifungals within one week of ERCP and a subgroup with acute cholangitis. The primary outcome was a composite of death and invasive candidiasis within one year. Secondary outcomes included death, invasive candidiasis, and rehospitalization. Inverse probability of treatment weighting (IPTW) was performed using baseline characteristics. Adjusted hazard ratios and odds ratios were calculated. Among 197 inpatients, 51 (25.9%) received antifungals. At one year, the primary outcome occurred in 23 of 51 patients (45.1%) receiving antifungal therapy and in 67 of 146 patients (45.9%) who did not; the IPTW-adjusted hazard ratio was 0.93 (95% confidence interval 0.69–1.27; *p* = 0.66). No significant differences were seen in the acute cholangitis subgroup (*n* = 117). In this study, antifungal therapy was not associated with improved survival, lower rates of invasive candidiasis, or fewer readmissions. Findings support a conservative, stewardship-oriented approach to managing Candida-positive bile cultures in the absence of invasive disease.

## 1. Introduction

*Candida* species (spp.) are commonly isolated from bile cultures obtained during endoscopic retrograde cholangiopancreatography (ERCP) [1], yet their clinical significance in this setting remains unclear. Despite a lack of evidence indicating pathogenicity, antifungal therapy is often initiated when *Candida* spp. are isolated from ERCP-obtained biliary cultures. While many *Candida* species are considered top priority pathogens by the World Health Organization [2], this practice raises concerns regarding overtreatment with antifungals and consequent increased drug resistance and toxicity. In an era of growing attention to antimicrobial stewardship, defining when antifungal therapy is warranted is increasingly prioritized [3,4,5,6,7].

At our institution, 28.5% of patients have *Candida* spp. in their ERCP-collected bile cultures [8], similar to the prevalence of 20–30% reported in other publications [1,9,10,11]. Although it is commonly encountered, current reports of clinical outcomes in these patients are limited to small and highly selected populations. For example, one retrospective study found an association between biliary *Candida* spp. isolation and reduced survival in patients with unresectable cholangiocarcinoma [11]; however, its generalizability and causal relationship remain unclear. Importantly, there is no published evidence that antifungal therapies in patients with *Candida* species-positive bile cultures improve outcomes.

To address this knowledge gap, a retrospective cohort study was undertaken to evaluate the clinical impact of antifungal therapy in patients with *Candida* spp. isolated from bile cultures obtained during ERCP. Outcomes assessed included death, invasive candidiasis, and rehospitalization at 30 days, 90 days, and one year. A subgroup analysis of patients with acute cholangitis was also conducted. This study aims to inform clinical decision-making and antifungal stewardship efforts by clarifying the utility of antifungal treatment in this setting.

## 2. Materials and Methods

### 2.1. Study Design and Inclusion Criteria

The study objectives were addressed using a retrospective cohort design including patients from Indiana University Health University Hospital, a tertiary referral center performing >3000 ERCPs per year. All adult patients (≥18 years) who underwent an ERCP between January 2010 and December 2023 with a bile culture positive for any *Candida* spp. were included. There were no patient exclusions based on the level of immunosuppression, comorbid conditions, or concomitant medications (apart from antifungals).

Patients were classified into the treatment group if they received at least one dose of systemic intravenous or oral antifungal therapy, with activity against the *Candida* spp. grown in culture, within seven days following the ERCP procedure from which the bile culture was obtained. Those who did not receive antifungal therapy within this timeframe were assigned to the control group. A seven-day window was selected to reflect standard clinical practice at our institution, during which antifungal decisions are typically made based on early culture results and clinical trajectory. The follow-up period was one-year to include patients who may have delayed or subacute onset of disease. Patients were excluded if they had invasive candidiasis (candidemia or deep-seated infection with *Candida* spp. by operative culture from a normally sterile site) within one year prior to the index ERCP date or if they had no follow-up records. The study was approved by the center’s institutional review board.

Medical records were used to retrospectively abstract demographics, past medical history, medication use, clinical data, ERCP reports, and clinical notes. Survival was determined through the medical record and the Database Registration of Indiana’s Vital Events (DRIVE). Data were abstracted by four of the authors via Research Electronic Data Capture (REDCap). To assess interrater reliability, a fifth blinded, unique author repeated an independent data abstraction on a random selection of 10% of patient records. Cohen’s kappa statistics and intraclass coefficients were calculated. This study’s reporting adheres to the Strengthening the Reporting of Observational Studies in Epidemiology (STROBE) guidelines.

### 2.2. Patient Identification

Patients were identified by cross-referencing a database of patients who underwent ERCPs with a list of patients with bile cultures positive for *Candida* spp. Collection of the bile culture by ERCP was confirmed during chart review. The technique used for bile aspiration and processing by our group has been previously described and is standardized across providers [12]. All cultures were collected at the discretion of the endoscopist based on clinical presentation and findings during ERCP.

### 2.3. Definitions

The index ERCP date was defined as the date on which the initial *Candida* species-positive bile culture was collected. This was used as the starting date to calculate all time-based outcomes. Invasive candidiasis was defined as the presence of *Candida* spp. in cultures from blood or surgically collected cultures from sterile spaces. Cultures from the respiratory tract, urinary tract, skin, and wounds were not considered due to high rates of colonization. Antifungal use was defined as at least one administration of an appropriate antifungal agent with activity against the relevant *Candida* spp. Acute cholangitis was defined as the presence of all three of the following: (1) fever (>38.0 °C) or hypothermia (<36.0 °C) within 72 h of the index ERCP, (2) any liver function test (total bilirubin, aspartate transaminase, alanine transaminase, or alkaline phosphatase) greater than 1.5 times the upper limit of normal at the Indiana University Health Pathology Laboratory, and (3) biliary obstruction confirmed during the index ERCP.

### 2.4. Outcomes

The primary outcome was a composite of invasive candidiasis or death within one year of the index ERCP. Secondary outcomes included rehospitalization, invasive candidiasis alone, and death alone. The subgroup of patients with acute cholangitis was similarly analyzed.

### 2.5. Statistical Analysis

The baseline characteristics of the included patients were compared via the chi- square test or Fisher’s exact test for categorical variables and the Mann–Whitney *U* test for continuous variables. To balance the baseline characteristics for the comparative analyses, an inverse probability of treatment weighting (IPTW) adjustment was applied by calculating a propensity score. The demographics and clinical variables used to calculate the propensity score included age, sex, race, body mass index (BMI), current smoking status, intensive care unit status, ERCP-performing physician, *Candida* spp., biliary disease (neoplasms, lithiasis, pancreatitis, primary sclerosing cholangitis), immunosuppressed status, acute cholangitis diagnosis (except when analyzing the subgroup of patients with acute cholangitis), biliary hardware in place at the index ERCP, and prior sphincterotomy.

A 30-day, 90-day, and one-year survival analysis was completed. To identify predictors of the primary outcome, a univariate logistic regression analysis was performed. When cell counts were zero, the Haldane–Anscombe correction method was used in order to calculate stable odds ratios and confidence intervals. The multivariate logistic regression analysis included the use of antifungals and baseline characteristics identified in the univariate analysis at a *p*-level < 0.05. Hazard ratios were calculated using a Cox proportional hazards model, and associated Kaplan–Meier curves were plotted. Sensitivity analyses were performed by excluding specific sub-cohorts of clinical relevance and performing the main analyses as outlined above, to ensure those groups were not driving the results. All analytic assumptions were verified, and analyses were performed using SAS v9.4 (SAS Institute, Cary, NC, USA).

## 3. Results

### 3.1. Patient Selection

**Figure 1:**
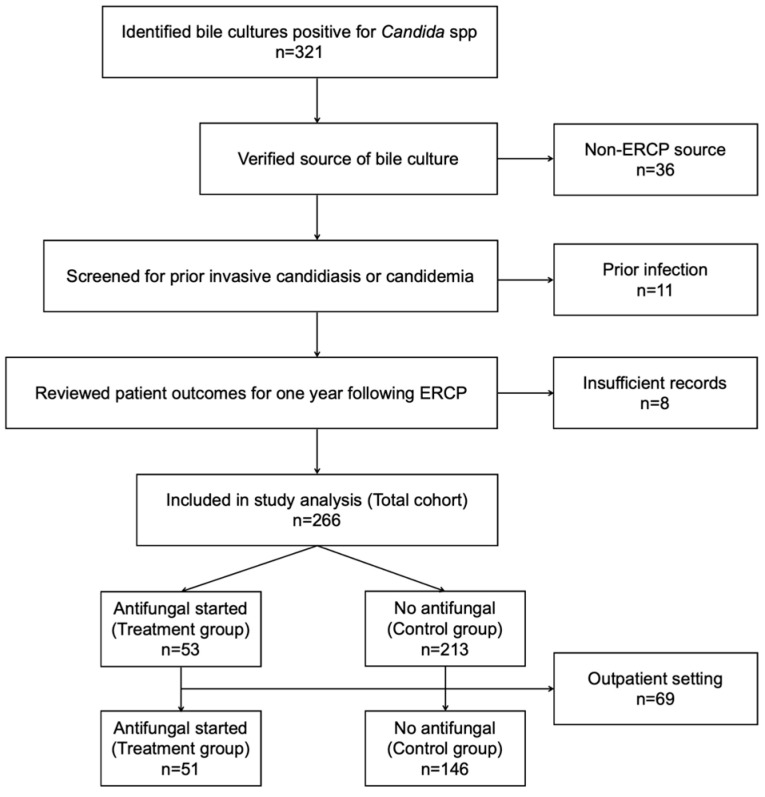
Study flowchart of patient selection with final group designations. Abbreviations: ERCP, endoscopic retrograde cholangiopancreatography.

A total of 321 potential patients were identified. Patients were excluded for the following reasons: bile culture not obtained via ERCP (*n* = 36, 11.2%), history of prior invasive candidiasis (*n* = 11, 3.4%), and insufficient records (*n* = 8, 2.5%). Among the 266 remaining patients, 53 (19.9%) received antifungal medication within one week of the index ERCP, and 213 (80.1%) did not (Figure 1). Antifungals administered within the first seven days of the index ERCP included fluconazole (86.8%) and micafungin (13.2%) at the adequate dosages with a duration of treatment ranging from 1 to 42 days, with a median of 7 days (9.8 ± 8.8, mean ± SD). Preliminary analysis of the total cohort (Supplemental Table S1) showed disproportionately higher antifungal use within seven days of index ERCP among inpatients compared to outpatients: 25.9% (51/197) of patients who underwent an inpatient ERCP received antifungal therapy compared to only 2.9% (2/69) of those who underwent an outpatient ERCP. As this imbalance suggested marked differences and possible bias in antifungal prescription due to severity of illness and ease of treatment implementation, we restricted the comparative analyses to inpatients only (*n* = 197).

**Figure 2:**
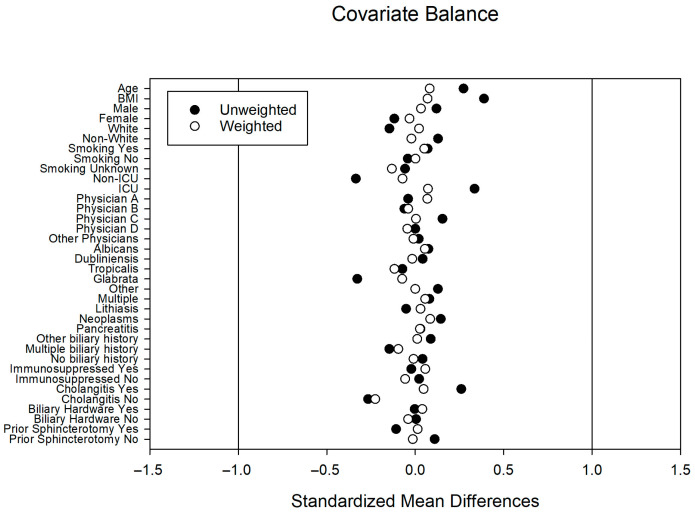
Love plot of standardized mean differences (SMD) before and after inverse probability of treatment weighting (IPTW) in the Inpatient Cohort.

Unadjusted and IPTW-adjusted baseline characteristics of the inpatients are reported in Table 1. Among the patients, 81.2% were White and 57.4% were male. Most patients (76.1%) had a known history of biliary disease, with 31% having previously diagnosed biliary neoplasms. The most frequently isolated species was *Candida albicans* (63.5%). Compared to those who did not receive antifungal therapy, patients treated within seven days of the index ERCP were more likely to be in the ICU at the time of the procedure (29.4% vs. 12.3%) and had a significantly higher BMI (30.5 vs. 27.7). Other baseline characteristics, including *Candida* spp., grown in bile culture, were not significantly different between the treatment and control groups. After IPTW adjustment, there was no longer an imbalance in ICU-level status, BMI, or other characteristics between groups (Table 1 and Figure 2).

### 3.2. Comparative Analysis

**Figure 3:**
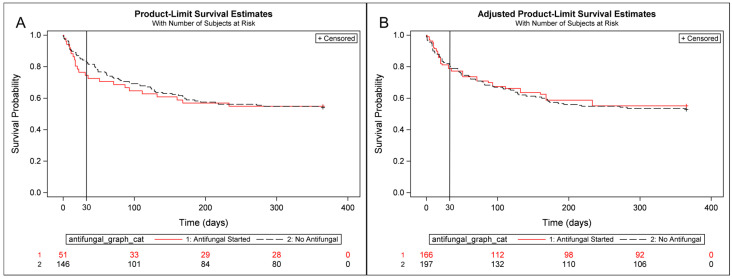
Kaplan–Meier curves of the primary outcome with number at risk for both (**A**) unadjusted and (**B**) IPTW-adjusted inpatient cohort (*n* = 197) comparing the treatment group and control group. Abbreviations: HR, hazard ratio; IPTW, inverse probability of treatment weighting; CI, confidence interval.

At 30 days, the primary outcome (composite of death and invasive candidiasis) occurred in 12 out of 51 patients (23.5%) in the treatment group and 23 out of 146 patients (15.8%) in the control group (Table 2). For those who received antifungals within seven days of the index ERCP, the unadjusted hazard ratio (1.65 [95% CI, 0.75–3.61]; *p* = 0.21) and IPTW-adjusted hazard ratio (1.06 (95% CI, 0.62–1.80, *p* = 0.84)) showed no statistically significant difference in the primary outcome between treatment groups as represented in the primary outcome survival curves (Figure 3). Antifungal treatment was not associated with a statistically significant difference in either 90-day or 1-year survival for the primary outcome, with IPTW-adjusted odds ratios (ORs) of 0.93 (95% CI, 0.59–1.45, *p* = 0.74) and 0.93 (95% CI, 0.69–1.27; *p* = 0.66), respectively (Supplemental Table S2).

Mortality at 30 days was 12 of 51 patients (23.5%) in the treatment group and 17 of 146 patients (11.6%) in the control group (IPTW-adjusted HR 1.42 [95% CI, 0.81–2.48]; *p* = 0.22). The unadjusted 30-day mortality showed that antifungal treatment was associated with significantly higher odds of death (OR 2.34 [95% CI, 1.03–5.31]; *p* = 0.043), which can be attributed to the higher acuity of treated patients. Importantly, the 1-year mortality was high across both groups at 23 of 51 patients (45.1%) of the treatment group and 60 of 146 patients (41.1%) of the control group (OR 1.18 [95% CI, 0.62–2.24]; *p* = 0.62). Invasive candidiasis developed within 30 days for zero patients in the treatment group and 6 patients (4.1%) in the control group. The associated Chi-square test did not reach statistical significance (*p* = 0.13), and the OR could not be calculated due to a zero group (Table 2). The IPTW-adjusted 90-day and 1-year analyses for mortality alone and invasive candidiasis alone showed no statistically significant difference (Supplemental Table S2).

One-year readmission rates between groups were not significantly different, with total readmissions in one year being 47.1% versus 48.6% for the treatment and control groups, respectively (Supplemental Table S2). Based on this, the OR for readmission within one year after receipt of an antifungal was 0.94 (95% CI, 0.50–1.78; *p* = 0.86). The 30-day and 90-day analyses, with and without IPTW adjustment, for readmission showed no statistically significant difference (Table 2 and Supplemental Table S2). Survival analyses for readmission, with and without IPTW adjustment, similarly showed no difference in patient trends (Supplemental Figure S1).

### 3.3. Impact of Antifungal Treatment Duration

To further evaluate the impact of antifungal therapy duration, we analyzed outcomes comparing patients who received short-course (≤7 days, *n* = 27) and long-course (>7 days, *n* = 23) against the control group (*n* = 146). When assessed over the entire study period, the duration of antifungal treatment did not significantly impact the primary outcome; the IPTW-adjusted odds ratio (OR) was 0.94 for both the short-course and long-course groups compared to the control group (*p* = 0.9065 and *p* = 0.8990, respectively).

However, when assessing specific early time intervals (30 and 90 days), the limited sample size and low event rate within the >7 days cohort precluded reliable adjusted modeling. Therefore, 30-day and 90-day comparative analyses were restricted to the ≤7 days group versus the control group. In this restricted analysis, the ≤7 days group exhibited higher mortality at both 30 days (OR 2.71; *p* = 0.0014) and 90 days (OR 1.88; *p* = 0.0205). Conversely, the ≤7 days group had significantly lower odds of readmission at 30 days (OR 0.45; *p* = 0.0460), 90 days (OR 0.40; *p* = 0.0059), and over the entire study period (OR 0.53; *p* = 0.0002). The >7 days group, evaluated only over the full study period, demonstrated significantly higher odds of readmission (OR 2.10; *p* = 0.0001).

### 3.4. Univariate and Multivariate Regression Analyses

A univariate logistic regression analysis was conducted to assess select demographic and clinical characteristics (Table 3). Patients had a significantly increased risk of reaching the primary outcome if they had known neoplasms (OR 5.53 [2.95, 10.39 95% CI]; *p* < 0.0001), biliary hardware present prior to the index ERCP (OR 2.56 [1.39, 4.69 95% CI]; *p* = 0.0025), or an immunosuppressed status at the time of the index ERCP (OR 2.40 [1.25, 4.60 95% CI]; *p* = 0.0083).

A multivariate logistic regression analysis was performed by including treatment with antifungals and all statistically significant variables from the univariate analysis. The presence of neoplasms remained a significant risk factor for occurrence of the primary outcome (OR 4.82 [2.34–9.94 95% CI]; *p* < 0.0001), but biliary hardware and immunosuppressed status were no longer significant risk factors; receipt of antifungals was also not associated with the primary outcome (Table 3).

### 3.5. Acute Cholangitis and Sensitivity Analysis

A subgroup analysis was conducted for members of the inpatient cohort meeting acute cholangitis criteria (*n* = 117) and similarly showed no statistically significant differences in any outcomes (Supplemental Tables S3 and S4). Survival analyses for the primary outcome and readmission, with and without IPTW adjustment, showed no difference in outcomes between treatment and control groups in the acute cholangitis subgroup (Supplemental Figures S2 and S3).

A sensitivity analysis was conducted by excluding patients with known neoplasms (*n* = 122) and similarly showed no statistically significant differences in outcomes (Supplemental Table S5).

### 3.6. Interrater Reliability Statistical Analysis

To assess interrater reliability, a blinded reviewer independently re-abstracted 10% of the records (*n* = 26). Agreement for categorical variables was generally high, with Cohen’s kappa ranging from 0.43 to 1.00. Perfect agreement (κ = 1.00) was observed for sex, *Candida* species, death, and readmission. Several variables, including antifungal use (κ = 0.88) and cholangitis diagnosis (κ = 0.92), showed strong agreement. Notably, some variables had moderate kappa values despite high raw agreement—such as lithiasis (κ = 0.43, 81%) and prior sphincterotomy (κ = 0.58, 79%)—likely due to imbalanced prevalence. Continuous variables showed excellent reliability, with intraclass correlation coefficients ranging from 0.94 to 1.00 (Supplemental Tables S6 and S7).

## 4. Discussion

In this retrospective cohort of inpatients with *Candida* spp. growth in ERCP-collected bile cultures, antifungal therapy was not associated with reduced mortality, invasive candidiasis, or rehospitalization at 30, 90, or 365 days. Results were consistent after inverse probability of treatment weighting (IPTW), in subgroup analyses of patients with acute cholangitis, and in sensitivity analyses excluding patients with neoplasms. Although multivariate analysis identified neoplasms as a risk factor for the composite outcome, antifungal therapy itself was not associated with improved outcomes.

Our findings support the hypothesis that isolation of *Candida* spp. from bile cultures collected during ERCP often represents colonization or contamination from the intestinal tract and does not require treatment. Importantly, invasive candidiasis, confirmed to be caused by the same species of *Candida* from the original ERCP bile culture, developed within 30 days in 6 patients (4.1%) who did not receive antifungal therapy compared to zero in the antifungal group. This was not a statistically significant finding, but it does highlight the potential value of a large, randomized controlled trial to further investigate this potential signal. Alternatively, if treatment were necessary, one would anticipate a significant mortality signal in the untreated group, given the well-documented high 30-day mortality rate (30–55%) associated with invasive candidiasis [13,14]. Instead, no differences in mortality were detected between groups despite a full year of follow-up. These results align with the established biological behavior of *Candida* spp. as largely non-pathogenic in non-sterile environments, while leaving open the possibility that antifungal therapy prevents invasive candidiasis in a small subset of patients. *Candida* spp. commonly colonize the gastrointestinal tract and can translocate to cause invasive disease in susceptible hosts [15,16,17,18]. Our results suggest that such progression is rare following ERCP, supporting that most biliary isolations represent colonization and do not require empiric or prophylactic therapy.

When interpreting the impact of treatment duration, it is important to consider the clinical dynamics of a retrospective cohort. While our primary IPTW analysis robustly adjusted for baseline severity regarding the decision to initiate antifungal therapy, stratifying by treatment duration introduces complex variables tied to a patient’s clinical trajectory. The higher early mortality observed in the short-course (<7 days) group likely reflects a subset of critically ill patients who unfortunately experienced early clinical decline, which naturally truncated their treatment course and precluded the possibility of readmission. Conversely, patients receiving prolonged therapy (>7 days) may represent a cohort that survived the acute phase of illness, thereby having a greater opportunity for subsequent readmission. Therefore, these duration-specific findings should not be interpreted as a failure of short-course therapy or an indication for prolonged treatment. Ultimately, the fact that the primary composite outcome did not differ significantly from untreated controls over the entire study period—regardless of whether a short- or long-course was administered—reinforces our conclusion that empiric antifungal therapy does not provide a clear overall survival benefit in this population. Positive cultures for *Candida* spp. from urine or respiratory tract specimens usually represent colonization rather than true infection [19,20,21,22,23,24,25]. Similarly, most biliary *Candida* isolations in our cohort likely represented colonization rather than infection. Current Infectious Diseases Society of America (IDSA) and the European Society of Clinical Microbiology and Infectious Diseases (ESCMID) recommend against routine antifungal treatment for asymptomatic candiduria or respiratory *Candida* spp. colonization [26,27]. Antifungal therapy may be considered in critically ill or profoundly immunosuppressed patients, though evidence is limited [25]. Although biliary cultures are not specifically addressed in the guidelines, our findings suggest that a similar conservative approach may be appropriate for most patients.

There is growing interest in antifungal stewardship, particularly in light of rising antifungal resistance and the global burden of candidemia [3,4,5,6,7]. Several studies have demonstrated that stewardship interventions can reduce antifungal use and resistance [28,29]. A 2023 systematic review of 41 antifungal stewardship programs found up to a 50% reduction in antifungal expenditures without adverse impact on mortality [30]. Our findings reinforce the importance of targeted antifungal stewardship, particularly in clinical scenarios such as *Candida* spp. isolation from bile culture, where a significant benefit of antifungal therapy is yet to be identified.

This study did have several limitations. While IPTW adjustment mitigates measured confounders, unmeasured confounders, particularly related to provider decision-making, remain. Bile culture collection is generally performed at the discretion of the endoscopist, and initiation of antifungals is largely driven by the primary management team. Another clear limitation of this study is that treatment assignment was not randomized, with sicker patients more likely to receive antifungals, therefore creating a substantial source of selection bias. A prospective, randomized trial would be needed to resolve these issues. However, through this investigation at our center, with an exceptionally high volume of ERCPs over a 13-year period demonstrates that generation of the necessary sample size would likely require significant multi-center efforts. Loss to follow-up is also a potential limitation, although the use of statewide death records was implemented to partially address this. Most patients in this cohort had underlying biliary stents (62.9%), prior to sphincterotomy (67.5%), or known biliary disease (76.1%) which may influence the colonization rate and limit generalizability to less treatment-experienced populations. Additionally, *C. albicans* predominated among isolates, limiting species-specific conclusions. Inclusion in the treatment group required only a single effective antifungal dose due to the uncertain necessary treatment duration in this setting. While the efficacy of antifungals was confirmed by MIC testing, some patients received short courses either due to quick clinical improvement, death, or other clinical decision-making. Importantly, for the 6 untreated patients who developed invasive candidiasis within 30 days of the principal ERCP, all received adequate subsequent antifungal therapy with only a single associated death. Overall, these findings are most applicable to patients with advanced biliary disease undergoing ERCP.

In summary, this retrospective cohort study found no strong evidence that antifungal treatment improves outcomes in patients with *Candida* spp. isolated from bile cultures collected during ERCP, even in the context of significant underlying biliary pathology, acute cholangitis, or malignancy. However, a possible protective effect against invasive candidiasis was observed, warranting prospective investigation. These findings support the interpretation that most cases represent colonization or contamination rather than true infection, paralleling established practices regarding other non-sterile anatomic sites. While our results provide important guidance for antifungal stewardship in this setting, prospective, multicenter studies are needed to further refine clinical decision-making and optimize stewardship strategies.

## Figures and Tables

**Table 1:** Inpatient cohort baseline characteristics before and after IPTW adjustment.

	Unadjusted				IPTW-Adjusted		
	Overall *n* = 197 (%)	Antifungal Yes (*n* = 51)	Antifungal No (*n* = 146)	*p*-Value	Antifungal Yes	Antifungal No	*p*-Value
Age	64.4 (15.7)	67.4 (14.5)	63.3 (16.0)	0.1117	66.5 (26.7)	64.7 (18.3)	0.4649
Sex							
Male	113 (57.4)	32 (62.8)	81 (55.5)	0.3664	59.6	57.6	0.7037
Female	84 (42.6)	19 (37.3)	65 (44.5)		40.4	42.4	
Race							
White	160 (81.2)	39 (76.5)	121 (82.9)	0.3133	82.2	81.2	0.8115
Non-white	37 (18.8)	12 (23.5)	25 (17.1)		17.8	18.8	
BMI	28.4 (7.3)	30.5 (7.2)	27.7 (7.2)	0.0191	29.0 (11.4)	28.3 (8.7)	0.5526
Smoking							
Yes	94 (47.7)	26 (51.0)	68 (46.6)	0.8429	52.6	49.4	0.4287
No	85 (43.2)	21 (41.2)	64 (43.8)		42.3	42.2	
Unknown	18 (9.1)	4 (7.8)	14 (9.6)		5.0	8.4	
Treatment location							
Inpatient (non-ICU)	164 (83.3)	36 (70.6)	128 (87.7)	0.0049	79.9	83.3	0.4017
ICU	33 (16.8)	15 (29.4)	18 (12.3)		20.1	16.7	
Performing physician							
A	73 (37.1)	18 (35.3)	55 (37.7)	0.7464	42.5	38.3	0.9411
B	47 (23.9)	11 (21.6)	36 (24.7)		21.8	23.8	
C	27 (13.7)	7 (13.7)	20 (13.7)		11.5	13.2	
D	9 (4.6)	4 (7.8)	5 (3.4)		4.5	4.4	
Others	41 (20.8)	11 (21.6)	30 (20.6)		19.8	20.3	
*Candida* species							
*C. albicans*	125 (63.5)	34 (66.7)	91 (62.3)	0.4742	67.9	64.7	0.8195
*C. glabrata*	26 (13.2)	3 (5.9)	23 (15.8)		10.6	13.3	
*C. dubliniensis*	10 (5.1)	3 (5.9)	7 (4.8)		4.5	4.9	
*C. tropicalis*	10 (5.1)	2 (3.9)	8 (5.5)		2.5	4.6	
Other	7 (3.6)	3 (5.9)	4 (2.7)		2.5	2.5	
Multi	19 (9.6)	6 (11.8)	13 (8.9)		12.1	10.0	
Biliary disease							
Neoplasms	61 (31.0)	19 (37.3)	42 (28.8)	0.6315	36.3	31.3	0.2651
Lithiasis	22 (11.2)	5 (9.8)	17 (11.6)		13.6	12.4	
Pancreatitis	14 (7.1)	4 (7.8)	10 (6.9)		7.7	6.9	
PSC	7 (3.6)	0 (0)	7 (4.8)		0	3.6	
Other	19 (9.6)	5 (9.8)	14 (9.6)		9.8	9.4	
Multiple	27 (13.7)	5 (9.8)	22 (15.1)		9.9	13.3	
None	47 (23.9)	13 (25.5)	34 (23.3)		22.7	23.2	
Immunosuppressed							
Yes	52 (26.4)	13 (25.5)	39 (26.7)	0.8646	29.9	26.7	0.4880
No	145 (73.6)	38 (74.5)	107 (73.3)		70.1	73.3	
Cholangitis diagnosis							
Yes	117 (59.4)	36 (70.6)	81 (55.5)	0.0586	62.1	59.2	0.5762
No	80 (40.6)	15 (29.1)	65 (44.5)		27.9	40.8	
Biliary hardware present							
Yes	124 (62.9)	32 (62.8)	92 (63.0)	0.9727	66.2	63.8	0.6308
No	73 (37.1)	19 (37.3)	54 (37.0)		33.8	36.2	
Prior sphincterotomy							
Yes	133 (67.5)	32 (62.8)	101 (69.2)	0.3984	67.8	67.0	0.8626
No	64 (32.5)	19 (37.3)	45 (30.8)		32.2	33.0	

Abbreviations: ICU, intensive care unit; BMI, body mass index; PSC, primary sclerosing cholangitis; ERCP, endoscopic retrograde cholangiopancreatography. Values are expressed as means (standard deviations) for continuous variables and frequencies (percentages) for categorical variables.

**Table 2:** Frequency of the primary outcome and secondary outcomes (death, invasive candidiasis, and readmission) for the inpatient cohort at 30 days.

	Treatment Group ^a^ (*n* = 51)	Control Group ^a^ (*n* = 146)	Chi-Square or Fisher’s *p*-Value	Odds Ratio (95% CI) *p*-Value	IPTW-Adjusted HR (95% CI) *p*-Value
Death or invasive candidiasis					
Yes (either)	12 (23.5)	23 (15.8)	0.2110	1.65 (0.75, 3.61)	1.06 (0.62, 1.80)
No (neither)	39 (76.5)	123 (84.3)		*p* = 0.214	*p* = 0.8377
Death					
Yes	12 (23.5)	17 (11.6)	0.0392	2.34 (1.03, 5.31)	1.42 (0.81, 2.48)
No	39 (76.5)	129 (88.4)		*p* = 0.043	*p* = 0.2212
Invasive candidiasis					
Yes	0 (0)	6 (4.1)	0.3420	<0.01 (<0.01, >999) ^b^	<0.01 (<0.01, >999)
No	51 (100)	140 (95.9)		*p* = 0.954	*p* = 0.9289
Readmission					
Yes	9 (17.7)	30 (20.6)	0.6458	0.83 (0.36, 1.89)	0.99 (0.59, 1.66)
No	42 (82.4)	116 (79.5)		*p* = 0.655	*p* = 0.973

Abbreviations: HR, Hazard ratio; CI, confidence interval. ^a^ Expressed as frequency (percentage). ^b^ Haldane–Anscombe correction applied due to 0 group.

**Table 3:** Univariate and multivariate logistic regression analysis of the primary outcome for the inpatient cohort.

	Primary Outcome Yes ^a^ (*n* = 90)	Primary Outcome No ^a^ (*n* = 107)	Univariate Model OR (95% CI)	Univariate Model *p*-Value	Multivariate Model OR (95% CI)	Multivariate Model *p*-Value
Sex						
Male (reference)	56 (49.6)	57 (50.4)	0.69 (0.39, 1.23)	0.2064		
Female	34 (40.5)	50 (59.5)				
BMI						
<30	62 (50.8)	60 (59.2)	1.74 (0.96, 3.12)	0.0661		
≥30 (reference)	28 (37.3)	47 (62.7)				
Neoplasms						
Yes	53 (70.7)	22 (29.3)	5.53 (2.95, 10.39)	0.0001	4.82 (2.34, 9.94)	<0.0001
No	37 (30.3)	85 (69.7)				
Albicans						
Yes	57 (45.6)	68 (54.4)	0.99 (0.55, 1.77)	0.9747		
No	33 (45.8)	39 (54.2)				
Immunosuppression						
Yes	32 (61.5)	20 (38.5)	2.40 (1.25, 4.60)	0.0083	1.62 (0.79, 3.32)	0.1912
No	58 (40.0)	87 (60.0)				
In-patient non-ICU	73 (44.5)	91 (55.5)	0.76 (0.36, 1.60)	0.4621		
ICU (reference)	17 (51.5)	16 (48.5)				
Biliary hardware present						
Yes	67 (54.0)	57 (46.0)	2.56 (1.39, 4.69)	0.0025	1.09 (0.52, 2.27)	0.8169
No	23 (31.5)	50 (68.5)				
Prior sphincterotomy						
Yes	65 (48.9)	68 (51.1)	1.49 (0.81, 2.74)	0.1966		
No	25 (39.1)	39 (60.9)				
Antifungal treatment						
Yes	23 (25.6)	28 (26.2)	0.97 (0.51, 1.84)	0.9222	0.94 (0.47, 1.90)	0.8689
No (reference)	67 (74.4)	79 (73.8)				

Abbreviations: BMI, body mass index; ICU, intensive care unit; OR, odds ratio; CI, confidence interval. ^a^ Expressed as frequency (percentage).

## Data Availability

The original contributions presented in this study are included in the article/Supplementary Materials. Further inquiries can be directed to the corresponding author.

## Supplementary Materials

The following supporting information can be downloaded at: https://www.mdpi.com/article/10.3390/jof12030208/s1, Table S1: Total cohort baseline characteristics; Table S2: Survival analyses of primary outcome and secondary outcomes for the inpatient cohort at 90 and 365 days; Table S3: Frequency of primary outcomes for Acute Cholangitis subgroup at 1 year; Table S4: Survival analyses for acute cholangitis subgroup; Table S5: Frequency of primary outcome and secondary outcomes for the sensitivity analysis (neoplasms excluded) at 1 year; Table S6: Cohen’s kappa values for categorical variables; Table S7: Intraclass correlation coefficient values for continuous variables; Figure S1: Survival curves for hospital readmissions (A) unadjusted and (B) IPTW adjusted comparing treatment and control groups in the inpatient cohort (*n* = 197); Figure S2: Survival curves for primary outcome (A) unadjusted and (B) IPTW adjusted comparing treatment and control groups in the acute cholangitis subgroup (*n* = 117); Figure S3: Survival curves for hospital readmissions (A) unadjusted and (B) IPTW adjusted comparing treatment and control groups in the acute cholangitis subgroup (*n* = 117).
