# Supplementary material for: Outcomes Following Antifungal Treatment for Candida Growth in Bile Cultures Collected During Endoscopic Retrograde Cholangiopancreatography
Source: J Fungi (Basel). 2026 Mar 14;12(3):208. doi: 10.3390/jof12030208 (PMC13027859; doi:10.3390/jof12030208)
Supplement: Supplementary file 1 [file jof-12-00208-s001.zip › jof-4163074-supplementary.pdf]

Supplementary Material

Supplemental Table S1. Total Cohort Baseline Characteristics

	Overall (n=266)	Antifungal Yes (n=53)	Antifungal No (n=213)	p-value
Age	63.6 (15.8)	66.3 (15.4)	63.0 (15.8)	.1707
Sex				.5062
Male	160 (60.2)	34 (64.2)	126 (59.2)	
Female	106 (39.9)	19 (35.9)	87 (40.9)	
Race				.7512
White	210 (79.0)	41 (77.4)	169 (79.3)	
Non-white	56 (21.1)	12 (22.6)	44 (20.7)	
BMI	27.9 (7.2)	30.4 (7.3)	27.3 (7.0)	.0060
Smoking				.9571
Yes	131 (49.3)	27 (50.9)	104 (48.8)	
No	108 (40.6)	21 (39.6)	87 (40.9)	
Unknown	27 (20.2)	5 (9.4)	22 (10.3)	
Treatment location				**<.0001**
Out-patient	69 (25.9)	2 (3.8)	67 (31.5)	
In-patient (non-ICU)	164 (61.7)	36 (67.9)	128 (60.1)	
ICU	33 (12.4)	15 (28.3)	18 (8.5)	
Antifungal used				<.0001
Fluconazole	46 (17.3)	46 (86.8)	0 (0)	
Micafungin	7 (2.6)	7 (13.2)	0 (0)	
None	213 (80.0)	0 (0)	213 (100)	
Candida species				.6619
Albicans	175 (65.8)	36 (67.9)	139 (65.3)	
Glabrata	30 (11.3)	3 (5.7)	27 (12.7)	
Dubliniensis	12 (4.5)	3 (5.7)	9 (4.2)	
Tropicalis	12 (4.5)	2 (3.8)	10 (4.7)	
Other	10 (3.8)	3 (5.7)	7 (3.3)	
Multi	27 (10.2)	6 (11.3)	21 (9.9)	
Bile culture results				.9921
Polymicrobial	241 (90.6)	48 (90.6)	193 (90.6)	
Candida species only	25 (9.4)	5 (9.4)	20 (9.4)	
Biliary disease				.9803
Neoplasms	94 (35.3)	19 (35.9)	75 (35.2)	
Lithiasis	25 (9.4)	5 (9.4)	20 (9.4)	
Pancreatitis	20 (7.5)	4 (7.6)	16 (7.5)	
PSC	12 (4.5)	2 (3.8)	10 (4.7)	
Other	27 (10.2)	5 (9.4)	22 (10.3)	
Multi	34 (12.8)	5 (9.4)	29 (13.6)	
None	54 (20.3)	13 (24.5)	41 (19.3)	
Immunosuppressed				.7987
Yes	74 (27.8)	14 (26.4)	60 (28.2)	
No	192 (72.2)	39 (73.6)	153 (71.8)	
Acute cholangitis diagnosis				.0003
Yes	132 (49.6)	38 (71.7)	94 (44.1)	
No	134 (50.4)	15 (28.3)	119 (55.9)	
Prior ERCP				.3228
Yes	209 (78.6)	39 (73.6)	170 (79.8)	
No	57 (21.4)	14 (26.4)	43 (213)	
Biliary hardware present				.3132
Yes	181 (68.1)	33 (62.3)	148 (69.5)	
No	85 (32.0)	20 (37.7)	65 (30.5)	
Prior sphincterotomy				.1449

Yes	192 (72.2)	34 (64.2)	158 (74.2)	
No	74 (27.8)	19 (35.9)	55 (25.8)	

Abbreviations: ICU, intensive care unit; BMI, body mass index; PSC, primary sclerosing cholangitis; ERCP, endoscopic retrograde cholangiopancreatography.

Values are expressed as means (standard deviations) for continuous variables and frequencies (percentages) for categorical variables.

Supplemental Table S2. Survival Analyses of Primary Outcome and Secondary Outcomes for the Inpatient Cohort at 90 and 365 Days.

		Antifungal treatment Yes (n=51)	Antifungal treatment No (n=146)	Odds ratio (95% CI) with p-value	IPTW adjusted OR (95% CI) with p-value
90 days	Primary Outcome				
	Yes (either)	17 (33.3)	43 (29.5)	1.20 (0.61, 2.37)	0.93 (0.59, 1.45)
	No (neither)	34 (66.7)	103 (70.6)	p=.604	p=.744
	Death				
	Yes	17 (33.3)	36 (24.7)	1.53 (0.76, 3.06)	1.16 (0.73, 1.83)
	No	34 (66.7)	110 (75.3)	p=.231	p=.528
	Invasive Candidiasis				
	Yes	0 (0)	9 (6.2)	<0.01 (<0.01, >999)	<0.01 (<0.01, >999)
	No	51 (100)	137 (93.8)	p=.944	p=.912
	Readmission				
	Yes	16 (31.4)	49 (33.6)	0.91 (0.46, 1.79)	1.14 (0.73, 1.76)
	No	35 (68.6)	97 (66.4)	p=.775	p=.569
1 year	Primary outcome				
	Yes (either)	23 (45.1)	67 (45.9)	0.97 (0.51, 1.84)	0.91 (0.60, 1.38)
	No (neither)	28 (54.9)	79 (54.1)	p=.922	p=.657
	Death				
	Yes	23 (45.1)	60 (41.1)	1.18 (0.62, 2.24)	1.09 (0.72, 1.65)
	No	28 (54.9)	86 (58.9)	p=.618	p=.694
	Invasive candidiasis				
	Yes	0 (0)	11 (7.5)	0.11 (0.01, 1.97)	<0.01 (<0.01, >999)
	No	51 (100)	135 (92.5)	p=.959	p=.937
	Readmission				
	Yes	24 (47.1)	69 (47.3)	0.99 (0.52, 1.88)	1.14 (0.76, 1.73)
	No	27 (52.9)	77 (52.7)	p=.980	p=.526

Values are frequencies (percentages). The Logistic regression odds ratios are for the antifungal treatment group having the event within the specified time window.

Supplemental Table S3. Frequency of primary outcomes for Acute Cholangitis subgroup at 1 year.

	Antifungal Yes (n=36)	Antifungal No (n=81)	χ² test p-value	OR (95% CI)
Death or invasive candidiasis				
Yes (either)	19 (52.8)	43 (53.1)	.9574	0.99 (0.45, 2.17);
No (neither)	17 (47.2)	38 (46.9)		p=.9754
Death				
Yes	19 (53.8)	40 (49.4)	.7346	11.5 (0.52, 2.51);
No	17 (47.2)	41 (50.6)		p=.7349
Invasive candidiasis				
Yes	0 (0)	6 (7.4)	.1752	<0.01 (<0.01, >999);
No	36 (100)	75 (92.6)		p=.9500
Readmission				
Yes (in- or out-patient)	17 (47.2)	36 (44.4)	.781	1.12 (0.51, 2.46);
No (neither)	19 (52.8)	45 (55.6)		p=780

Values are frequencies (percentages) with p-values from chi-square tests (Fisher's Exact where necessary). Logistic Regression Unadjusted Odds Ratio given with 95% confidence intervals and p-values.

Supplemental Table S4. Survival Analyses for Acute Cholangitis subgroup.

	HR (95% CI)	p-value
Primary outcome unadjusted	1.07 (0.62, 1.83)	.8151
Primary outcome with IPTW adjustment	1.02 (0.72, 1.45)	.9080
Readmission unadjusted	1.00 (0.57, 1.78)	.9880
Readmission with IPTW adjustment	1.13 (0.76, 1.69)	.5426

Values are hazards ratios (95% confidence intervals) for primary outcome (death or invasive candidiasis development) and hospital readmission, when having the antifungal treatment, from proportional hazard survival analyses.

Supplemental Table S5. Frequency of Primary Outcome and Secondary Outcomes for the Sensitivity Analysis (neoplasms excluded) at 1 year.

	Antifungal Yes (n=31)	Antifungal No (n=91)	χ^2 test p-value	OR (95% CI)
Death or invasive candidiasis				
Yes (either)	9 (29.0)	28 (30.8)	0.856	0.92 (0.38, 2.25); p=.856
No (neither)	22 (71.0)	63 (69.2)		
Death				
Yes	9 (29.0)	21 (23.1)	0.506	1.36 (0.55, 4.41) p=.507
No	22 (71.0)	70 (76.9)		
Invasive candidiasis				
Yes	0 (0)	9 (9.9)	0.110	<0.01 (<0.01, >999); p=.945
No	31 (100)	82 (90.1)		
Readmission				
Yes (in- or out-patient)	15 (48.4)	38 (41.8)	0.520	1.31 (0.58, 2.96); p=.236
No (neither)	16 (51.6)	53 (58.2)		

Values are frequencies (percentages) and logistic regression odds ratios.

Supplemental Table S6. Cohen’s Kappa Values for Categorical Variables

Variable Group	Variable	Kappa	Agreement (%)
	Sex	1	100
Race	White	0.8354	96
	Black	1	100
	Asian	1	100
	Race other	n/a	96
	Smoking history	0.7313	88
	History of known biliary disease	1	100
Biliary disease	Neoplasms	1	100
	Lithiasis	0.4348	81
	Pancreatitis	0.8354	96
	Primary sclerosing cholangitis	1	100
	Other	0.6232	92
	History of prior ERCP	1	100
	Treatment Location	0.8194	92
Candida species	Albicans	1	100
	Glabrata	1	100
	Dubliniensis	1	100
	Tropicalis	1	100
	Lustianiae	1	100
	Additional microorganism detected	1	100
	Antifungal started within one week	0.8824	96
	Long term prior antibiotic use	1	100
	Immunosuppression	0.6929	88

	Biliary Hardware	0.8	88
	Prior Sphincterotomy	0.5833	79
	Cholangitis diagnosis	0.9222	96
Cholangitis criteria	Fever within three days	0.7826	90
	Hypothermia within three days	0.7851	92
	Biliary obstruction at ERCP	n/a	94
	Alkaline phosphatase >187.5	0.8976	96
	ALT >78	0.9202	96
	AST >58.5	0.7607	88
	Bilirubin >1.5	0.8194	92
Outcomes	Death within one-year	1	100
	Invasive candidiasis within one-year	n/a	100
	Inpatient readmission within one-year	1	100

Based on duplicated chart abstraction (n=26) by a unique data collector.
Abbreviations: ERCP, endoscopic retrograde cholangiopancreatography; ALT, alanine aminotransferase; AST, aspartate aminotransferase.

Supplemental Table S7. Intraclass Correlation Coefficient Values for Continuous Variables

Variable	ICC
Weight	0.99695
Height	0.99957
BMI	0.99664
Additional micro number	0.94065
Patient age	1

Based on duplicated chart abstraction (n=26) by a unique data collector.
Abbreviations: BMI, body mass index; ICC, intraclass correlation coefficient.

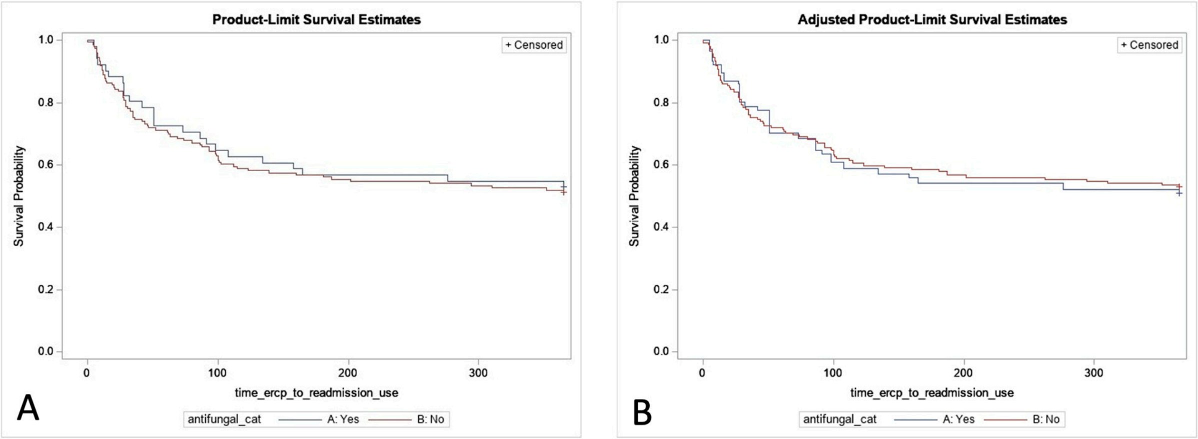

Supplemental Figure S1. Survival curves for hospital readmissions (A) unadjusted and (B) IPTW adjusted comparing treatment and control groups in the inpatient cohort (n=197)

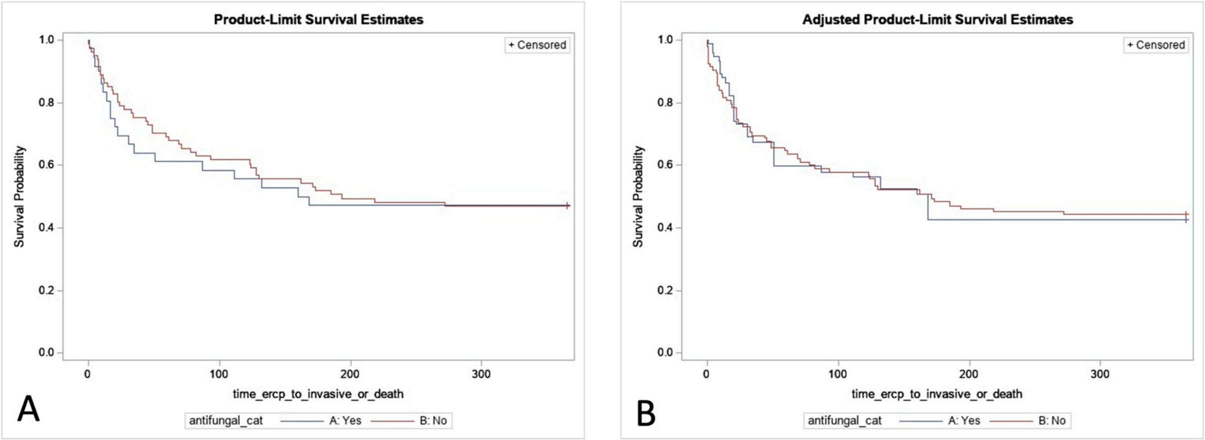

Supplemental Figure S2. Survival curves for primary outcome (A) unadjusted and (B) IPTW adjusted comparing treatment and control groups in the acute cholangitis subgroup (n=117)

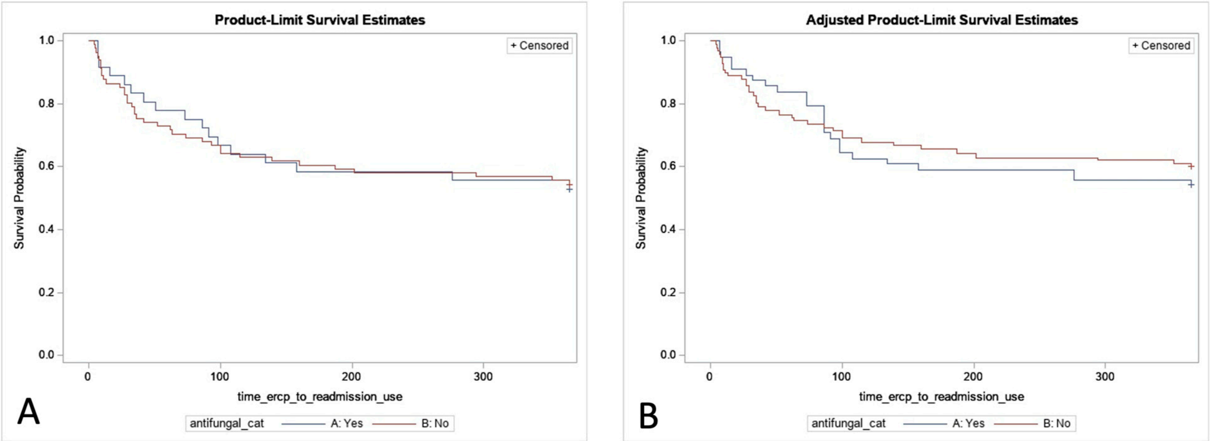

Supplemental Figure S3. Survival curves for hospital readmissions (A) unadjusted and

(B) IPTW adjusted comparing treatment and control groups in the acute cholangitis subgroup (n=117)
